# Enhanced Recovery After Surgery (ERAS) in Spine Surgery: A Systematic Review and Meta-Analysis of Spinal Surgery Sub – Specialities, Interventions and Efficacy

**DOI:** 10.1177/21925682251393697

**Published:** 2025-11-06

**Authors:** Caroline Büchel, Caroline Treanor, Benjamin Davies, David B. Anderson, Michael Fehlings, Carl M. Zipser, on behalf of the AO Spine – RECODE DCM ERAS Committee

**Affiliations:** 1 Spinal Cord Injury Center and Department of Neurology and Neurophysiology, Balgrist University Hospital, Zurich, Switzerland; 2 The School of Physiotherapy, Dublin 2 and The Department of Physiotherapy & The Department of Neurosurgery, Beaumont Hospital, 8863 Royal College of Surgeons in Ireland, Dublin, Ireland; 3 Division of Neurosurgery, Department of Clinical Neurosciences, 2152 University of Cambridge, Cambridge, UK; 4 School of Health Sciences, Faculty of Medicine and Health, 4538 University of Sydney, Sydney, NSW, Australia; 5 Division of Neurosurgery and Spinal Program, University of Toronto and Krembil Brain Institute, 7989 University Health Network, Toronto, ON, Canada

**Keywords:** enhanced recovery, enhanced recovery after surgery, ERAS, perioperative care, spine surgery

## Abstract

**Study Design:**

Systematic Review.

**Objectives:**

Enhanced Recovery After Surgery (ERAS) is a widely acknowledged approach for improving surgical outcomes. This review aims at analyzing the characteristics of study populations, interventions and outcomes in spine patients.

**Methods:**

Embase and Ovid were searched from inception until March 2025. We followed PRISMA guidelines. Study quality and risk of bias were assessed. In addition to a narrative synthesis of the evidence, a meta-analysis of RCTs evaluating length of stay (LOS) for a lumbar spine population was conducted. This review was registered prospectively on PROSPERO (No. CRD42025638293).

**Results:**

1431 records were identified, from which 81 studies were included. Reports of ERAS predominantly exist for degenerative spine pathologies (n = 35/81, 43.2%) and spinal deformities (n = 29/81, 35.8%). Most studied interventions were postoperative analgesia, early mobilisation (both n = 61/81, 75.3%) and patient education (n = 60, 74.1%). The most frequently used outcome measures were LOS (n = 65/81, 92.9%) and complication rates (n = 40/81, 49.4%). The overall median complication rate for ERAS patients was found to be lower (8.8% vs 15.6%). There was a statistically non-significant tendency for ERAS shortening LOS for 1 day in lumbar spine patients [95%CI -2.77, 0.71; *P* = 0.25].

**Conclusions:**

ERAS in spine surgery appears to be effective in terms of reducing LOS and complication rates. Further efforts at refining pain management and targeted disease-specific interventions are required. Whether ERAS interventions applied to individuals with significant neurological impairment and/or medical frailty, can influence surgical outcomes needs to be further studied.

## Introduction

There are a number of specific spinal structural pathologies for which elective spine surgery is a recommended therapeutic option. This includes severe or progressive spinal deformity, moderate to severe degenerative cervical myelopathy, persistent disabling radicular leg or arm pain secondary to lumbar or cervical disc herniation and disabling neurogenic claudication secondary to moderate or severe lumbar spinal stenosis. With the aging population, the demand for degenerative spinal surgery is growing and the frequency and complexity of comorbidities is increasing.^1,2^ Enhanced Recovery After Surgery (ERAS) pathways are a multidisciplinary approach that involves interventions throughout the entire surgical journey, from pre-operative preparation to post-operative care, with the goal of reducing complications, readmissions, and length of stay. Typical ERAS pathways consist of a collection of evidence-based, multimodal perioperative interventions designed to modulate surgical stress and achieve early recovery for patients undergoing major surgery.^3-5^ Over the past years, several surgical disciplines have successfully implemented ERAS into their clinical routine. For example, in the field of hepatobiliary surgery, an ERAS society guideline was published in 2016.^6^ Based on its success,^7^ the guidelines for perioperative care in liver surgery where updated in 2022^8^ and published together with a complementary ERAS society guideline dedicated to perioperative care for liver transplantation.^9^ The distinguishing factor ensuring excellence of ERAS society guidelines is that recommendations are made by a panel of leading experts based upon current evidence. Published guidelines are re-examined for updates at a two- to three-year interval.^10^ To date, in the field of spine surgery, there has only been one ERAS society guideline published which was specific to patients undergoing lumbar fusion surgery, although a number of research teams have investigated the effectiveness of locally developed ERAS pathways in single institutions. This study aims to provide an overview of the spine pathology groups in which ERAS pathways have been implemented. It also aims to describe the ERAS interventions included and outcomes investigated in ERAS pathways which have been implemented in the field of spine surgery to date. If feasible, this study also aims to investigate the effectiveness of ERAS pathways in spine surgery in reducing length of stay. By narrowing ERAS to spine surgery, insights shall be advisory for stakeholders willing to implement an ERAS pathway into their clinical routine in spine surgery as well as informing ERAS pathway developers regarding the current state of the art.

## Material and Method

### Eligibility Criteria

Study inclusion was based upon screening of title and abstract. There were no limits set regarding publication date. Studies were included if they contained reports on ERAS interventions in spine surgery. To be included, articles had to be available in English and of primary research (ie, no reviews, conference abstracts, etc.).

### Information Sources

Databases searched were Embase and OVID between June 22^nd^ 2024 until March 27^th^ 2025.

### Search Strategy

The search strategy was developed with the aid of a medical librarian. We designed a search algorithm using MeSH – terms related to spine surgery, spinal pathologies and ERAS – related keywords. Due to study inclusion being based on the screening of title and abstract, we limited our search to studies using these keywords either in their title and/or abstract.

### Data Collection Process

Our search algorithm yielded a total of 1431 studies. After deduplication, 1235 studies were left for screening. The same authors (CZ, CB) who had independently screened titles and abstracts for study inclusion were also responsible for data extraction and evaluation of study quality. We did not rely on any artificial intelligence, neither to obtain nor to interpret data. Since this is a systematic review, there was no need to recruit participants nor to obtain ethical approvement. This systematic review was prospectively registered on PROSPERO (No. CRD42025638293).

### Data Items

Studies were grouped according to study design, methodological quality, outcomes and comparisons and assessed for heterogeneity. Outcome comparison was done based on availability of data, ie, the most often used measure, for instance length of stay in days, would be used for further analysis. In order to assess reproducibility of ERAS protocol components, the five most frequently implemented interventions were rated from level one to three. To be considered as a level one intervention, description information regarding timing, format and content had to be provided, for level two information concerning timing or format had to be given and for level three there used to be no further clarification of the intervention.

Quality assessment was done using the National Heart, Lung and Blood Institute (NHLBI) tool. Study quality and risk of bias was rated separately to account for methodological differences.^11^

### Study Risk of Bias Assessment

Risk of bias was independently assessed by two authors (CZ, CB) using the ROBINS-I-V2 tool.

### Effect Measures

The median difference was used to synthesize and present findings of this review. For meta-analysis, we chose a 95% confidence interval to assess effect estimates. Results were visually presented using a forest plot.

### Synthesis Methods

Studies that were judged to be adequately homogenous and of good methodological quality were used for meta-analysis. If studies assessed the same outcome using different measures and/or at different (multiple) time points, the outcome measure and timepoint most frequently used were selected. Statistical analysis for our meta-analysis was done using SPSS.^12^ For studies that were too heterogeneous, a structured narrative summary following the SWiM reporting guidelines was used to synthesize the quantitative effect when meta-analysis of effect sizes was not feasible.^13^

## Results

### Overview of Study Design and Selected Outcome Measures

**Figure 1.**
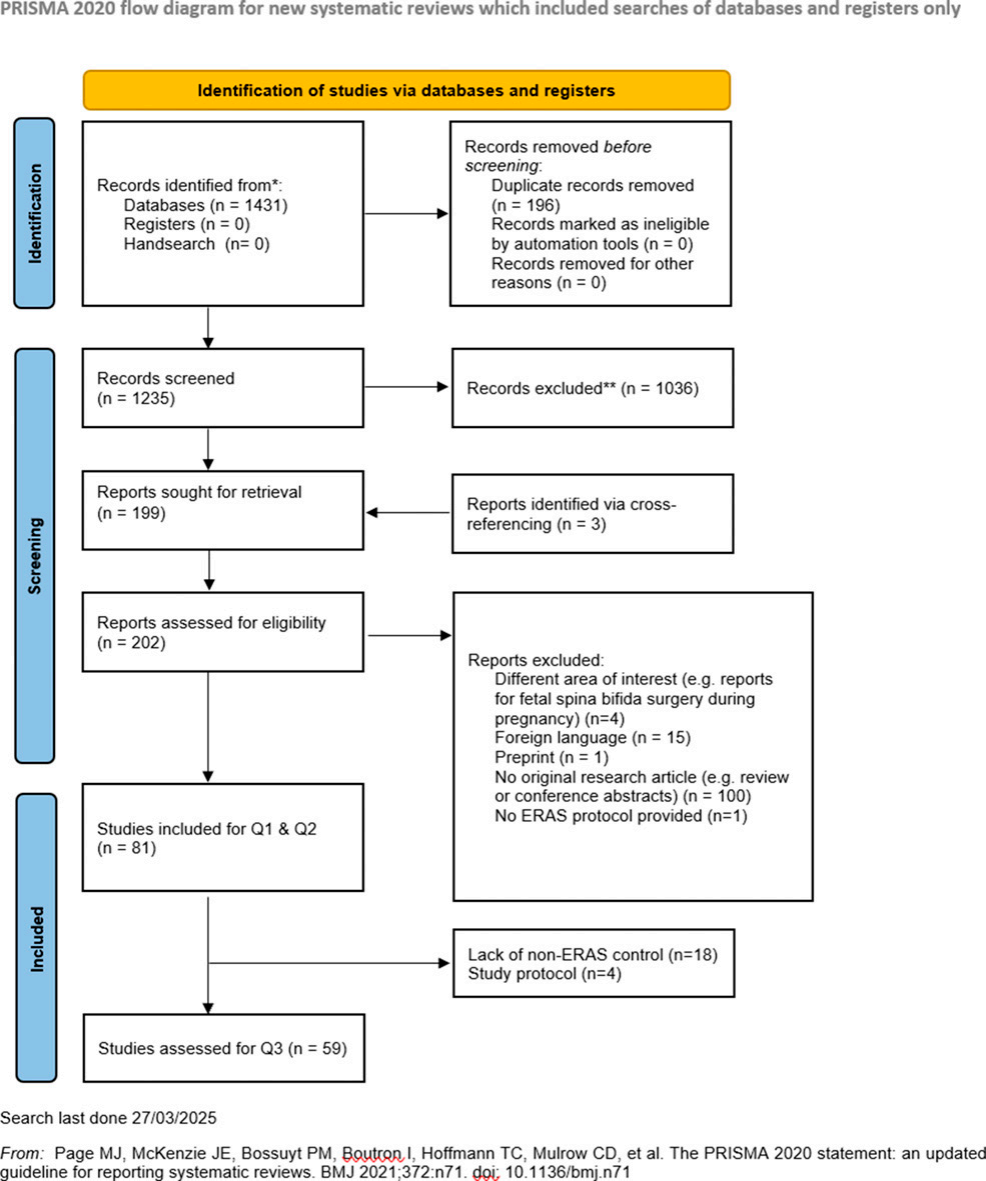
PRISMA Flowchart for Study Inclusion

**Figure 2.**
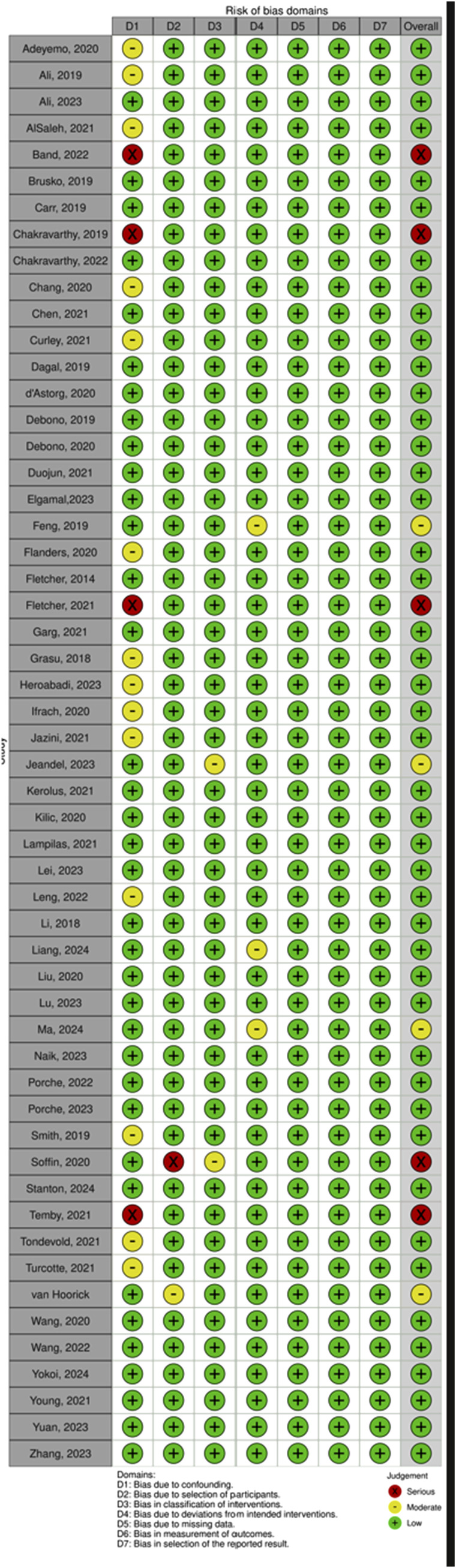
Risk of Bias as Determined Using the RoB 2 Tool

Our search algorithm yielded a total of 1431 records (Figures 1 and 2; PRISMA) and after deduplication and screening of title and abstract, 199 studies were retrieved for closer evaluation. Main reasons for article exclusion were (1) no original research article such as systematic reviews or (2) foreign language. Three articles were additionally retrieved via cross-referencing.^14-16^ Eventually, a total of 81 studies were included, with a majority being cohort studies (n = 62/81, 76.5%), followed by RCTs (n = 7/81, 8.6%),^17-23^ case series (n = 6/81, 7.4%)^24-29^ and study protocols (n = 4/81, 4.9%).^30-33^ Risk of bias for studies comparing an ERAS cohort with traditional or conventional care (TDC) was low in 81.4% (n = 48/59). Studies without a non-ERAS control group were not assessed in terms of Q3 (n = 22/81, 27.2%) The majority of studies were rated to be of good or fair quality (n = 43/77, 55.8% resp. N = 28/77, 36.4%). Most frequently studied outcome domains were LOS (n = 65/81, 92.9%), complication rates (n = 40/81, 49.4%) and postoperative pain (n = 39, 48.1%). In general, implementation of ERAS into clinical routine appears to be feasible and is associated with decreased LOS (n = 56/60, 93.3%), complication rates (median of 8.8% vs 15.6%) and costs (n = 15/16, 93.8%).

### Types of Spinal Surgery and Levels of the Spine Studied

**Figure 3.**
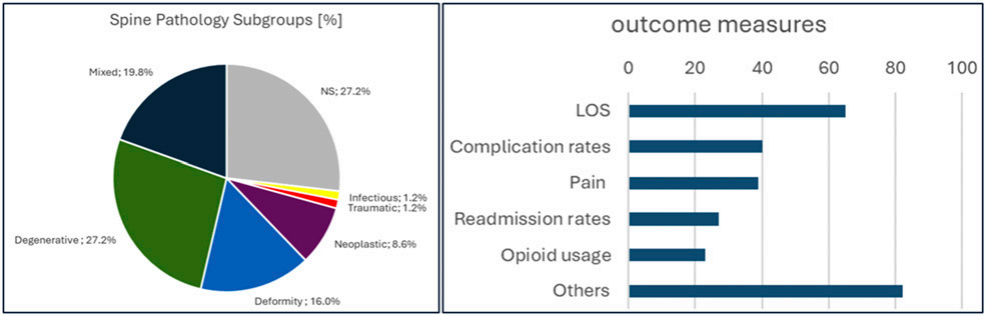
(A) Share of Spine Pathology Subgroups and (B) Outcome Domains Assessed by Studies (Multiple Outcome Measures Possible)

Most articles addressed the use of ERAS in the context of degenerative spine pathologies (n = 35/81, 43.2%), followed by deformity (n = 29/81, 35.8%). Only one study included patients with vertebral or spinal trauma into their study population.^34^ Various studies investigated a broader population with spinal pathologies with mixed spine pathology subgroups (n = 16/81, 19.8%), eg, patients suffering from both degenerative and deformative disease.^27,34-48^ Spinal fusion surgery was the surgical procedure being the most frequently investigated surgical technique used by ERAS protocols (n = 61/81, 75.3%). In terms of spinal regions, the lumbar spine (n = 56/81, 69.1%), followed by the cervical spine (n = 30/81, 37%) were the most frequently involved treatment sites. Of note, the same ERAS protocol was frequently applied for multiple types of surgeries (n = 42/81, 51.9%) and/or spinal regions (n = 34/81, 42%). Type of surgery was unclear in five studies (6.2%), while the underlying spinal disease requiring surgical treatment was unclear in 27.2% (n = 22/81).^18,19,22,25,26,30,49-65^ This may be due to ERAS pathways being tailored to the particular surgical intervention(s) studied rather than the disease-specific patient population. Nine studies (11.1%) studied the applicability and effectiveness of ERAS in the context of paediatric spinal surgery for adolescent idiopathic scoliosis,^14-16,66-69^ neuromuscular^70^ or congenital scoliosis (Figure 3).^23^

### ERAS Interventions Implemented

**Figure 4.**
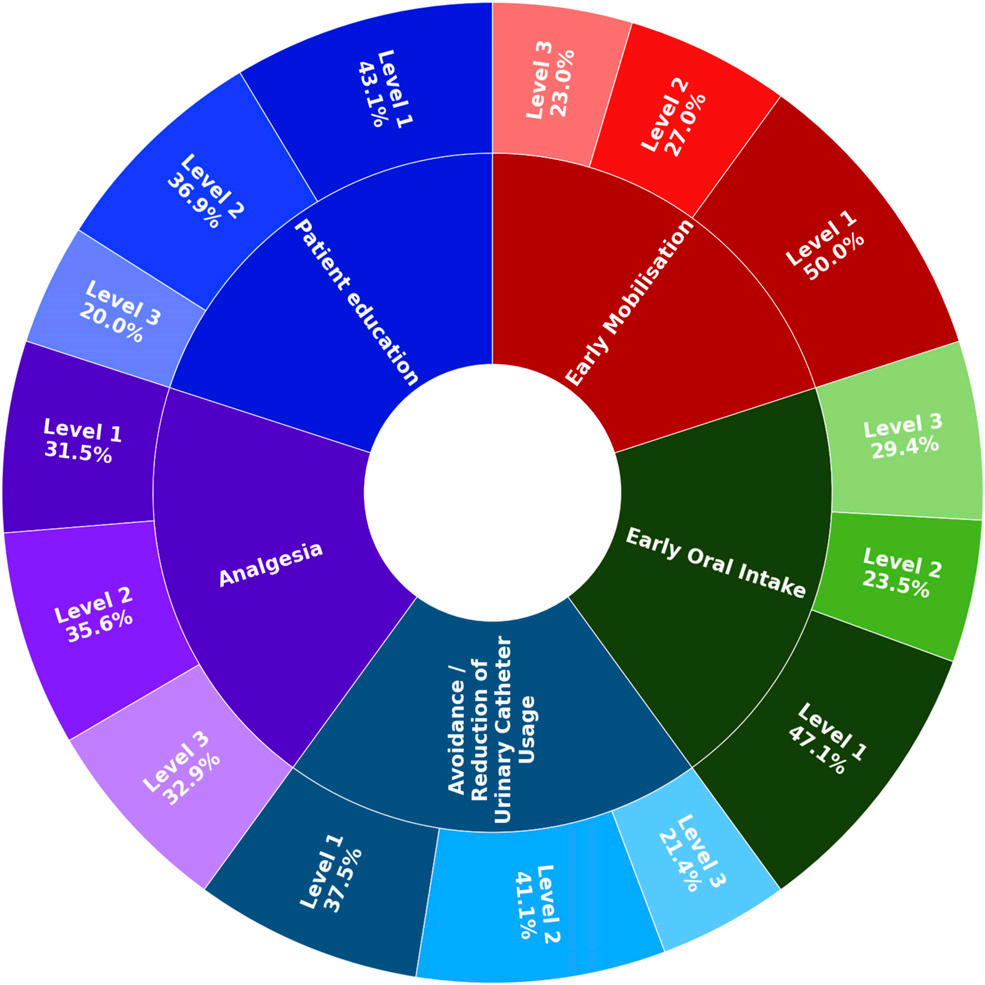
Level of Intervention Reporting for the Five Most Commonly Implemented ERAS Interventions (Level 1 = Information About Timing, Format and Content, Level 2 = information About Timing or Format, Level 3 = no Information Provided, Only Key Word Used)

The median number of interventions implemented was 12. Interventions most commonly used were patient education (n = 60/81, 74.1%), avoidance or reduction of urinary catheter usage (n = 51/81, 63%), postoperative analgesia and early mobilisation (both n = 61/81, 75.3%) and early oral intake (n = 49/81, 60.5%). Specificity in characterising ERAS interventions, eg, provision of detailed information upon dosing or type of analgesia, varied. We classified the aforementioned interventions into three different levels to account for the level of detail provided (Level 1 allowing reproduction of the trial findings to Level 3 providing few or no details at all). Early mobilisation was the most commonly precisely defined ERAS intervention, with 45.7% studies (n = 37/74) being considered as Level 1. These studies report how many hours after surgery and specify the type of mobilisation, ie, such as reporting whether patients were asked to sit up, ambulate, etc (Figure 4).

### Outcome Measures Investigated

Sixty studies (74.1%, n = 81) compared LOS between a traditional care and an ERAS cohort. Of these, 56 (93.3%, n = 60) reported a shorter LOS for the ERAS group. Protocol components used in more than half of all articles reporting a shortened LOS with ERAS were patient education (n = 42/60, 70%), early mobilisation (n = 42/60, 70%), postoperative analgesia (n = 40/60, 66.7%), avoidance/reduction of urinary catheter usage (n = 37/60, 61. 7%), multimodal analgesia (n = 34/60, 56.7%), early oral intake (n = 34/60, 56.7%) and antimicrobial prophylaxis (n = 32, 53.3%). The median number of interventions used did not differ between articles reporting an in- or decreased LOS.

**Figure 5.**
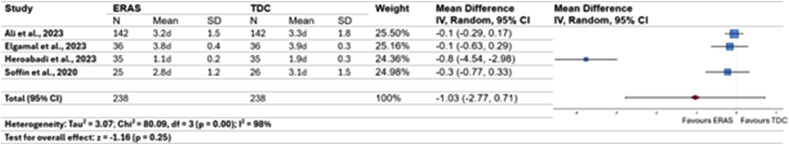
LOS Comparing LOS of Patients Suffering From Lumbar Spine Conditions Undergoing ERAS vs. TDC Care

To further assess potential effects of ERAS on LOS, we conducted a meta-analysis using an inverse-variance approach (Figure 5). For this, we included the four RCTs studying ERAS for patients undergoing elective lumbar spine surgery.^17-19,22^ Since Soffin et al. reported patients’ LOS using median and interquartile ranges as measure,^22^ we converted these data assuming a normal distribution of data points in order to interpret their findings along with the other studies. In general, length of stay was shown to be 1 day shorter [95% CI -2.77, 0.71] for patients undergoing ERAS care, yet did this result not reach statistical significance (*P* = 0.25). We waived any further statistical considerations for lack of study populations homogenous enough for comparison using similar outcome measures.

A trend towards a decrease in complication rates was visible. The median complication rate for ERAS patients was 8.8% lower than for patients undergoing TDC (15.6%) This was especially pronounced in the case of genitourinary complications (median complication rate of 2.5% for ERAS vs median of 5.4% for TDC) and incidence of postoperative delirium (complication rate of 2.2% ERAS vs 5.4% TDC). Only ten studies (25%) would report an altogether increased complication rate for their ERAS cohort in comparison to the TDC cohort.^16,57,59,62,65,66,71-74^ Several articles did not account for complication rates^15,17,26,29,37,44,50,51,60,63,64,69,75-83^ or would only provide an inconclusive amount of data, eg, only numbers for SSI^52^ or ‘adverse reactions/events.’^20,27^

Significant heterogeneity in reporting pain scores was observed. Thirteen studies using the Visual Analog Scale (VAS) at one day after surgery (POD1) were identified. Of these, seven (53.9%, n = 13) reported lower pain scores in the ERAS group,^22,23,49,67,72,84,85^ 6 (46.2%, n = 13) reported higher pain scores.^43,50,59,80,86,87^ Generalisability of this finding is difficult, since patient populations, types of surgery and applied ERAS interventions were diverse.

A cost analysis was conducted for 16 studies (19.8%, n = 81).^16,21,34,38,41,42,48,51,53,60,61,64,67,68,84,88^ Total hospital costs were reduced in 15 reports (93.8%, n = 16), with a median reduction of total costs of 1161.95$. Upon calculating costs per day, a median cost increase of 350.12$ was observed in ten articles (62.5%, n = 16). Studies reporting lower costs per day did not include reduction of urinary catheter usage and application of tranexamic acid and were also less likely to include patient education or early mobilisation into their ERAS protocol.

## Discussion

### Summary of Main Findings

There are a number of positive reports on multicomponent ERAS interventions reducing LOS, complications, and costs mostly in degenerative and deformity spine surgery. Patient cohorts were commonly stratified according to the surgical procedure and not the spinal diagnosis. For postoperative pain, which is a key priority for patients, a clear benefit from ERAS interventions was not reported; however, neither were negative impacts reported. Hence, ERAS appears to be an efficient tool to improve outcomes of patients undergoing spine surgery – and more importantly – feasible for implementation into clinical routine. What sets this systematic review apart is its focus on characterization of the particular ERAS interventions. High levels of details allow better reproducibility and may be of guidance for stakeholders willing to implement an ERAS care protocol at their institution.

### Reporting of ERAS Intervention Details

A precise description of ERAS interventions is a requirement to reproduce trial findings and to implement these interventions at other institutions. Despite a significant number of studies that would seamlessly allow implementation in clinical trials or implementation, a considerable number of studies did not provide sufficient detail. The highest level of detail for describing the particular interventions in spinal surgery were observed for early mobilisation (n = 37/74, 50%) and patient education (n = 28/65, 43,1%). For instance, Ifrach et al. advocated mobilisation aided by the nursing team within 6 hours after surgery and provided recommendations for frequency and timing of ambulation.^59^ Lei et al., educated patients using both health education manuals and videos, thereby already introducing patients to rehabilitation exercises.^89^

For antimicrobial prophylaxis, half of the studies reported either the type of antibiotic used or the timing of application. However, complete information was only available in 23.9% (n = 11/46) of studies. A potential explanation for this is that antimicrobial prophylaxis often follows clinical and pharmacological standards, ie, recommended dosing in specific patient population depending on kidney function and tolerance. Where antimicrobial prophylaxis is being conducted by eg, preoperative antibiotic solution skin swabs, exact data about dosing may then be difficult to determine. Therefore, details of antimicrobial prophylaxis might be omitted in certain cases. However, even if details are not provided, it is still useful to have information about the source of information used for deciding on drugs and dosages, ie, internal SOP or guidelines.

Of note, it cannot be excluded that some of the studies in this review included ERAS interventions that were not explicitly mentioned. This is related to the interventions being previously regarded as ERAS study interventions (eg, anaemia management, smoking cessation) but having gradually been implemented into clinical routine.^43^ Hence, the exact number of intervention inclusion into ERAS protocols may be higher, respectively inclusion of certain classical ERAS items may not always have been reported. This would align with findings from Echt et al. for lumbar spinal fusion, who found that ERAS components as well as intervention combination varied over time. However, this variance diminished from 2013 – 2016 in comparison to 2006 – 2012.^56^ This suggests that either some sort of consensus for the definition of ERAS is establishing itself or that variations in clinical routines between institutions are diminishing.

Additionally, it should be noted that only 31 studies (38.3%) included recommendations for surgical techniques into their ERAS care protocol. This could be explained by the fact that the same ERAS care protocol may have been implemented for different surgical procedures, ie, ERAS was introduced for certain surgical procedures rather than surgical procedure being adapted to an ERAS protocol. We found that most studies used ERAS for populations undergoing different types of fusion surgery (n = 61/81, 75.3%). Only forty-one studies (50.6%) investigating a population undergoing the same surgical procedure. Furthermore, while only five studies (6.2%) did not explicitly inform about the type of surgery used, as many as 22 studies (27.2%) did not report the underlying spinal pathology being treated. We assume that this difference may be related to factors such as some patients having unclear or multiple diagnoses, and that the particular diagnosis may not be of relevance for enrolment into the study cohort in case the same or similar treatment is required. For retrospective studies, it may have also been easier to search their databases for types of surgery rather than diagnosis.

### Interrelation Between LOS Reduction and Economic Impact of ERAS

The noted decrease in LOS in over ninety percent of studies advocates ERAS as a tool for optimising healthcare efficiency in spinal surgery. Numerous authors have posited that the effect of ERAS is not only caused by the ERAS interventions themselves but also previously defined discharge criteria.^19,73,86^ Of note, only three studies of a total of fifty-five studies reported a longer LOS.^61,77,87^

Even though this may suggest ERAS as a powerful tool to discharge patients much earlier in comparison to TDC patients, the reduction in length of stay did not reach statistical significance in our meta-analysis. We explain this by the fact that there was considerable heterogeneity (I^2^ = 98%) which will have substantially biased our results.^90^ Although studies had a similar number of ERAS care pathway components, their protocols were neither identical nor were the same surgical techniques used. Besides, Ali et al. and Heroabadi et al. both included non-lumbar patients into their study cohorts.^17,19^ Another main limitation is that LOS was not a primary outcome measure in any of these studies. In order to obtain robust data allowing further interpretation of the interrelation between length of stay and ERAS, more studies addressing comparable patient populations and using more similar ERAS care protocols - potentially with power analyses to determine the effect size for showing statistically significant results for LOS – would be desirable to overcome this limitation. With only four RCTs with differing primary outcome measures and surgical techniques, drawing any final conclusions is not possible.

Implementation of ERAS may also be of economic interest given the rising cost of surgery.^91^ By reducing both LOS and complication rate, ERAS has been shown to lower total hospitalisation costs. From the set of studies evaluated, only one study found higher costs for the propensity - matched control group undergoing ERAS care, due to longer LOS.^61^

As observed by Fiasconaro et al.,^34^ a higher number of ERAS interventions allocated appear to be associated with reduced LOS, which underscores the utility of a comprehensive perioperative approach. However, our data indicates that the number of interventions is only one factor for positive clinical outcomes. This is of relevance since even though the number of interventions allocated to patients may be higher when undergoing ERAS care, hospitalisation costs are still lowered. The concept of improved and accelerated regaining of functional recovery may be even more significant when considering discharge dispositions. Previous studies have noted a trend towards higher home discharge disposition rather than skilled nursing facilities or comparable healthcare settings for ERAS patients.^30,37,43,85^ Hence we posit that even though ERAS protocols may appear as more time-consuming and resource-demanding than TDC, it is ultimately efficient in terms of clinical and economical outcomes.

While lowering LOS and expenses is of interest for all stakeholders, this must not be at the cost of an increased readmission or complication rate.^92^ We observed a promising overall reduction of complications with ERAS. We assume that the reduction in genitourinary complications is partly due to the avoidance, respectively reduction, of urinary catheter usage and potentially earlier mobilisation. This is of interest since genitourinary complications, in particular urinary tract infections, have also been identified as significant contributor for prolonged LOS and higher readmission rates.^93^ Lovecchio et al.,^94^ found that higher complication rates and delayed ambulation are among factors associated with prolonged LOS after long-level fusions for adult spinal deformity.

### Pain Management

As mentioned earlier, the descriptions of the ERAS interventions are poor. While a total of seventy-three studies reported including multimodal and/or postoperative analgesia into their ERAS protocol, only twenty-three (34.5%) provided the highest level of descriptions (ie, reported type of medication, dosing and administration intervals). In general, our results align with the findings of Dietz et al., who observed pain levels being reduced or at least stable with ERAS.^95^ This indicates that even though patient mobilisation tended to take place earlier and analgesia was more frequently following an opioid-sparing regime, pain levels were at least not increased. However, pain management remains challenging and further data and research are required to optimise patient experience of post-operative pain after spinal surgery. Thus, higher reporting level for interventions is critical to reproduce findings. Even more, distinguishing neuropathic and nociceptive pain is desirable, since they are caused by different underlying pathomechanisms. This suggests that they may benefit from distinct treatment approaches.

### Tailoring ERAS to Complex Patient Populations

The applicability and efficacy of ERAS interventions depends on the functional status of a patient. While solid evidence for ERAS exists for the treatment of patients primarily suffering from primarily orthopaedic conditions, more studies including more vulnerable patient populations with eg, neurological impairment or frail patients is of interest. Despite different aetiology, both may challenge essential features of ERAS such as avoidance of urinary catheters, early mobilisation or early oral intake. For instance, a patient with reduced mobility before surgery might be less susceptible to an “early mobilisation” scheme. This said, there is need to first discuss evidence from existing studies investigating complex populations with frailty and neuromuscular disorders.

Frail patients have been showed to be at risk for postoperative adverse events and worse postoperative recovery.^45^ Benefit from proactive patient management involving interventions such as optimisation of chronic illnesses and/or nutritional management as pursued by ERAS is likely to be high. Previous studies have shown that ERAS is also effective in treatment in difficult patient populations such as elderly patients.^33,40,45,59,87,96-100^ Similarly, patients with neuromuscular^70^ or congenital^101^ scoliosis have been effectively treated under guidance of ERAS protocols.

Interestingly, Tøndevold et al.,^82^ found decreased blood loss and shortened LOS for a neuromuscular scoliosis population when compared before and after implementation of ERAS for patients suffering from adolescent idiopathic scoliosis attended by the same ward, even though the protocol for neuromuscular scoliosis patients was not altered. The effects of ERAS introduction related to the harmonisation and reinforcement of interdisciplinary patient care was thus shown to be beyond protocol. This indicates that consciousness, ie, education levels and mindset of caregivers may play an at present underestimated role in the quality of care delivery.

Consequently, these findings pave the way for investigating the applicability of ERAS to patient populations on the edge of neuromuscular and frail. Degenerative cervical myelopathy (DCM) is typically a diagnosis of patients with advanced age.^102^ At the same time, patients suffering from moderate to severe DCM present with neurological impairment.^103-105^ This makes them a unique and even more demanding patient population likely to benefit from patient-centred care pathways such as ERAS, while at the same time challenging classical ERAS approaches such as avoidance of urinary catheters or early oral intake, etc. Development of a disease-tailored ERAS care protocol could be a substantial aid for tackling this issue.

### Strengths and Limitations

This systematic review is novel for its comprehensive investigation of ERAS strategies in spinal surgery. Therefore, it allows to compare different protocols over a variety of spinal pathologies and identification of key interventions along with core strengths and weaknesses of ERAS. Even though our meta-analysis indicated reductions in LOS as statistically non-significant, we may not forget that our results are biased by considerable heterogeneity, particularly in terms of surgical techniques utilised. While we can state that ERAS works for a diverse patient population, ranging from paediatric to geriatric patients, determination of age-specific care demands is impossible. Similarly, different populations and types of surgery are in relation to disparate complications. This issue was further intensified by diverse level of complication reporting. At this point, we also need to discuss possible publication bias since positive results may be more likely to be published than less satisfactory data. Conclusions regarding the efficacy of ERAS upon postoperative pain were insofar not feasible as that patient population, analgesic management as well as pain measurement varied heavily. Regarding the fact that monitoring interventions closely is a mean of creating bias, it is difficult to distinguish between the effect of intervention exposure and outcome assessment. Especially evaluation of pain is considered to have an inherent predisposition of being compromised by this so-called Hawthorne Effect. Lastly, monitoring pathway compliance and evaluation of reasons for non-adherence should not be neglected.

## Conclusion

Enhanced Recovery after Surgery, also known as ERAS, is an innovative multicomponent evidence-based approach to surgical care which appears to demonstrate significant benefit in spine surgery. Most evidence exists for ERAS implementation into degenerative and deformity surgery and is focused upon reducing LOS and complication rates while optimising pain management. ERAS interventions effectively improve outcomes and lower costs. There is need for further investigation of personalised ERAS recommendations based upon spine diagnosis such as DCM. Furthermore, despite being frequently studied, the assessment of pain management approaches remains challenging, in part due to variability and lack of precision in outcomes assessments.

## Supplemental Material

Supplemental Material - Enhanced Recovery After Surgery (ERAS) in Spine Surgery: A Systematic Review and Meta-Analysis of Spinal Surgery Sub – Specialities, Interventions and Efficacy Supplemental Material for Enhanced Recovery After Surgery (ERAS) in Spine Surgery: A Systematic Review and Meta-Analysis of Spinal Surgery Sub – Specialities, Interventions and Efficacy by Caroline Büchel, Caroline Treanor, Benjamin Davies, David B. Anderson, Michael Fehlings, Carl M. Zipser, on behalf of the AO Spine – RECODE DCM ERAS Committee in Global Spine Journal.

## Appendix

### Abbreviations

**ERAS**: Enhanced Recovery After Surgery, a multimodal perioperative approach aiming at improved patient recovery by re-examining conventional care
LOS: Length of Stay
TDC: traditional or conventional care
VAS: Visual Analog Scale
POD1: postoperative day 1
ACDF: anterior cervical discectomy and fusion
TLIF: transforaminal lumbar interbody fusion
SSI: surgical site infection
