# Supplementary material for: Enhanced Recovery After Surgery (ERAS) in Spine Surgery: A Systematic Review and Meta-Analysis of Spinal Surgery Sub – Specialities, Interventions and Efficacy
Source: Global Spine J. 2025 Nov 6;16(3):1660–72. doi: 10.1177/21925682251393697 (PMC12592135; doi:10.1177/21925682251393697)
Supplement: Supplemental Material - Enhanced Recovery After Surgery (ERAS) in Spine Surgery: A Systematic Review and Meta-Analysis of Spinal Surgery Sub – Specialities, Interventions and Efficacy [file sj-pdf-1-gsj-10.1177_21925682251393697.pdf]

| Author, Year [Number of Patients] | Study Design, Control Group | Indication for Surgery | Type of Surgery | Length of Stay | Pain Score Evaluation (Yes/No) | Complications (n, %) | Study quality[#] |
|---|---|---|---|---|---|---|---|
| **Adeyemo et al., 2020 [124]** | Cohort study with retrospective control | adult degenerative scoliosis | open thoraco-lumbar-pelvic fusion | 7d ± 3.88 | Yes | 14 (20.9%) | Good |
| | | | | 5.82d ± 1.97 | | 49 (85.96%) | |
| **Ali et al., 2019 [275]** | Cohort study with retrospective control, prospectively collected data | NS; «elective spine or peripheral nerve surgery" | laminectomy+/-fusion, discectomy+/-fusion, foraminotomy, thoracolumbosacral fusion, "other" | 3.6d ± 2.4 | Yes | 22 (10.9%) | Fair |
| | | | | 4d ± 3.2 | | 12 (16.2%) | |
| **Ali et al., 2018** | Study protocol | NS | NS | - | - | - | |
| **Ali et al., 2023 [284]** | RCT | NS | laminectomy+/-fusion, discectomy+/-fusion, thoracolumbosacral fusion, osteophyte resections, tumour resections, | 3.2d ±1.5 | Yes | Not reported | Fair |
| | | | | 3.3 ± 1.8 | | | |
| **Alsaleh et al., 2021 [93]** | Cohort study with retrospective control | scoliosis, degenerative lumbar stenosis, lumbar spondylolisthesis | thoracolumbar fusion surgery (PLF, TLIF, posterior fusion) | 4.2d±1.5 | Yes | 2 (5%) | Fair |
| | | | | 5.9d±3.3 | | 7 (14%) | |
| **Band et al., 2022 [32]** | Cohort study with retrospective control, prospectively collected data | Degenerative spine disease | MIS-TLIF | 1.6d | No | Not reported | Fair |
| | | | | 2.4d | | | |
| **Brusko et al., 2019 [97]** | Cohort study with retrospective control | NS | Posterior 1- to 3-level lumbar fusion surgery (open and MIS fusion) | 2.9 ± 1.9d | Yes | Not reported | Good |
| | | | | 3.8d ± 1.8 | | | |
| **Carr et al., 2019 [932]** | Cohort study with retrospective control (TDC vs. ERAS), concurrent control (NPC vs ERAS) | NS | anterior/posterior surgery, ≥ 4-level nonrevision surgery, ≥ 3-level revision surgery, revision anterior surgery, expected duration >6 hours, expected blood loss > 1 L, planned staged surgery, corpectomy, pedicle subtraction osteotomy (copied) | 5.4d | No | Not reported | Good |
| | | | | 8.2d (TDC)<br>8d (NPC) | | | |
| **Chakravarthy et al., 2019 [1770 / 156]** | Cohort study with retrospective control | NS | (Micro-)Discectomy ± fusion,, decompression ± fusion, tumour corpectomy / debulking, pedicle subtraction osteotomy | Not reported | No | SSI: 16 (2%) | Poor |
| | | | | | | SSI: 40 (4.12%) | |

| | | | | | | | |
|---|---|---|---|---|---|---|---|
| **Chakravarthy et al., 2022 [390]** | Cohort study with retrospective control | metastatic spine tumour | stabilisation &/ decompression (open and MIS) | 5.4d ± 5.6 | No | Not reported | Good |
| | | | | 7.5d ± 7.6 | | | |
| **Chan et al., 2017 [74]** | Prospective cohort study, no control group | adolescent idiopathic scoliosis | Posterior spinal fusion | 86.2 ±14.4hours | Yes | 1 (1.4%) | Fair |
| **Chang et al., 2020 [48]** | Cohort study with retrospective comparative control | degenerative lumbar spine disease | TLIF (ERAS cohort)<br>MIS-TLIF (TDC) | 1.4 ± 1.13 d | No | 1 (4.17%) | Fair |
| | | | | 4.0±1.98 d | | 0 (0%) | |
| **Chen et al., 2021 [78]** | Cohort study with concurrent control | disc herniation / stenosis / spondylolysis with main degeneration at L4/5 | TLIF (ERAS)<br>OLIF (TDC) | 9.23d ± 0.95 | Yes | Not reported | Poor |
| | | | | 7.87d ± 1.04 | | | |
| **Chen et al., 2024 [180]** | Study protocol | intradural extramedullary spinal tumours | *"All procedures under general anaesthesia for microchannel or vertebroplasty surgery"* | Yes | Yes | Yes | |
| **Choi et al., 2024 [88]** | Cohort study with retrospective control | Lumbar degenerative disease (degenerative or isthmic spondylolisthesis, spinal stenosis, neural foraminal stenosis) | One- or two-level posterior lumbar fusion | 5.23d (±0.87) | Yes | 2 (4.3%) | Good |
| | | | | *Postoperative Day 5.59d (±1.07)* | | 8 (19.5%) | |
| **Curley et al., 2021 [99]** | Cohort study with retrospective control | stenosis, myelopathy, spondylolisthesis, instability | PCDF | 2.5d ± 1.6 | No | Not reported | Fair |
| | | | | 2.9d ± 1.2 | | | |
| **Dagal et al., 2019 [558]** | Cohort study with retrospective control (TRDC) / concurrent control (NOPW) | NS | «elective major spine surgery» | 6.1d ± 3.6 | No | 28 (10.5%) | Good |
| | | | | TRDC: 8.2d ± 6.3<br>NOPW: 7.6d ± 5.1 | | TRDC: 23. (12.6%)<br>NOPW: 8 (7.4%) | |
| **d'Astorg et al., 2020 [386]** | Cohort study with retrospective controls, prospectively collected data | lumbar stenosis, disc herniation | microdiscectomy, arthrodesis, fusion surgery | 2.6d (median 2d, range 1-7) | Yes | Not reported | Good |
| | | | | 4.4d (median 4d, range 2-14) | | | |
| **De Bie et al., 2021 [60]** | Cohort study with retrospective control | NS | lumbar surgery (laminectomy, posterolateral fusion with anterior | Not reported | Yes | (complications related to | Fair |

| | | | | | | | |
|---|---|---|---|---|---|---|---|
| | | | interbody cage | | | morphine consumption: 13 (43.3%)) | |
| | | | | | | (30 (100%)) | |
| **Debono et al., 2019 [3483]** | Cohort study with retrospective controls, prospectively collected data | NS | fusion surgery (ACDF, ALIF, posterior fusion) | ALIF: 3.33d ± 0.8 / ACDF: 1.3d ± 0.7 / PSF: 4.8d± 2.3 | Yes | 194 (*30.5%*); of these, 67 (12.2%) were considered as major complications | Fair |
| | | | | ALIF: 6.06 ± 1.1 / ACDF: 3.08d ± 0.9 /PSF: 6.7d ± 4.8 | | 161 (*32.7%*); of these, 62 (7.4%) were considered as major complications | |
| **Debono et al., 2020 [539]** | Cohort study with retrospective controls, prospectively collected data | cervical radiculopathy with disc prolapse | ACDF | 1.40d ± 0.6 | No | 14 (6.9%) | Fair |
| | | | | 2.96d ± 1.35 | | 10 (5.0%) | |
| **Duojun et al., 2021 [120]** | Cohort study with retrospective control | lumbar disc herniation | discectomy | 3.47d ± 1.14 | Yes | 6 (10%) | Good |
| | | | | 5.65d ± 1.39 | | 9 (15%) | |
| **Echt et al., 2022 [132’351]** | Cohort study with Retrospective controls (Top ERAS 2006-2012 vs. Top ERAS 2013-2016) | NS | Posterior lumbar fusion | Median 2 (2-3) | No | 1286 (7.5%) | Good |
| | | | | Median 3 (2-3) | | 4883 (9.7%) | |
| **Elgamal et al., 2023 [72]** | RCT | NS | Lumbar decompression surgery | 3.83d ± 0.38 | Yes | 5 (13.9%) (PONV) | Fair |
| | | | | 3.89d ± 0.32 | | 11 (30.6%) PONV | |
| **Feng et al., 2019 [74]** | Cohort study with retrospective control | lumbar stenosis, spondylolisthesis, degenerative lumbosacral with instability / radiculopathy / neurogenic claudication | MIS-TLIF | median 5 (3-15) | No | 2 (4.55%) | Good |
| | | | | median 7 (5-12) | | 4 (13.3%) | |
| **Fiasconaro et al., 2020 [265'576]** | Cohort study with retrospective controls | vertebral / spinal cord trauma, malignancy, | posterior lumbar fusion | Median 3 (IQR 2.4) | No | 2033 (5.77%) | Good |

| | | | | | | | |
|---|---|---|---|---|---|---|---|
| | | deformity | | | | | |
| | | | | Median 3 (IQR 2.4) | | 17176 (7.46%) | |
| **Flanders et al., 2020 [1290]** | Cohort study with retrospective control | NS | laminectomy +/- fusion, foraminotomy, discectomy+/-fusion,, , peripheral nerve procedures | 3.4d ± 2.4 | Yes | 202 (17.7%) | Fair |
| | | | | 3.9d ± 2.5 | | 24 (16.1%) | |
| **Fletcher et al., 2014 [269]** | Cohort study with retrospective control | adolescent idiopathic scoliosis | Posterior spinal fusion | 2.92d ± 0.71 | No | 15,59% | Good |
| | | | | 4.28d ± 1.08 | | 10,4% | |
| **Fletcher et al., 2021 [276]** | Prospective dual-center study (ERAS vs. TDC) | adolescent idiopathic scoliosis | Posterior fusion | 2.2d (IQR: 2.1-2.3) | Yes | 3 *(1.47%)* | Good |
| | | | | 4.8d (IQR: 4-5.1) | | 1 *(1.37%)* | |
| **Garg et al., 2021 [812]** | Cohort study with retrospective control | lumbar disc herniation +/- instability, spondylolisthesis, stenosis, degenerative disc disease +/- instability, facet joint cyst | TLIF (open or MIS) | 2.94d ± 1.6 | Yes | 37 (11.7%) | Good |
| | | | | 3.68d ± 1.8 | | 67 (13.5%) | |
| **Gondar et al., 2024 [100]** | Consecutive case series | degenerative thoracolumbar spine disease | Thoracolumbar fusion surgery ± decompression | 7d ± 3.0 | Yes | 7 (7%) (requiring revision surgery) | Good |
| **Grasu et al., 2018 [97]** | Cohort study with retrospective control | metastatic spine tumour | decompression, stabilisation ± vertebrectomy, transcutaneous vertebroplasty ± stabilisation | 6.3 ± 2.2 d | Yes | 13 (31.7%)** | Good |
| | | | | 6.8d ± 1.9 | | 10 (17.9%)** | |
| **Han et al., 2024 [333]** | Cohort study with retrospective control, prospectively collected data | Disc herniation, spinal stenosis, spondylolisthesis, “others” | One- or two-segment TLIF | Elderly: 5.84d ± 3.03 Younger : 5.86d ± 3.39 | Yes | Elderly: 28 (24.8%) Younger: 20 (20%) | Fair |
| | | | | 7.22d ± 3.42 | | 45 (37.5%) | |
| **Heroabadi et al., 2023 [70]** | RCT | NS | laminectomy | 25.37 ± 3.87 hours | Yes | 0 (0%) | Fair |
| | | | | 46.63 ± 6.99 hours | | 0 (0%) | |
| **Howard et al., 2023 [3’449]** | retrospective cross-sectional analysis (white vs. BIPOC) | “elective spine or peripheral nerve surgery” | Laminectomy ± fusion, discectomy ± fusion, foraminotomy, peripheral nerve procedure, “other” | 3.4d ± 2.7 | Yes | 445 (15.5%) | Good |
| | | | | 3.8d ± 3.2 | | 79 (13.7%) | |
| **Huang et al., 2024 [233]** | Cohort study with retrospective control, | Degenerative disc disease, spinal stenosis | Multi-level posterior (thoraco-)lumbar fusion | 11.67d ± 7.26 | No | 41 (25.2%) | Fair |

| | | | | | | | |
|---|---|---|---|---|---|---|---|
| | prospectively collected data | | | | | | |
| | | | | 14.89d ± 7.78 | | 22 (31.4%) | |
| **Huang et al., 2025 [215]** | Cohort study with retrospective control | Thoracolumbar deformity | Multi-level (minimum 5 levels) deformity correcting fusion surgery, incl. laminectomy | 11.13d ± 4.16 | Yes | 33 (29.2%) | Good |
| | | | | 13.09d ± 4.57 | | 53 (52%) | |
| **Ifrach et al., 2020 [564]** | Cohort study with retrospective control | NS | cervical/thoracic/lumbar laminectomy and/or instrumented fusion, (ACDF), combined anterior-posterior surgeries, tumour resections peripheral nerve procedures | 3.7d ± 2.4 | Yes | 102 (20.2%) | Good |
| | | | | 4.3d ± 2.5 | | 10 (16.7%) | |
| **Jazini et al., 2021 [85]** | Cohort study with retrospective control | Adult spinal deformity | Lumbar fusion surgery | 5.2d ± 2.2 | Yes | Not reported | Fair |
| | | | | 5.8d ± 1.5 | | | |
| **Jeandel et al., 2023, [60]** | Cohort study with retrospective control | adolescent idiopathic scoliosis | posterior fusion surgery | 5d±0.9 | Yes | 1 (3.33%) | Good |
| | | | | 6d±0.9 | | 1 (3.33%) | |
| **Kerolus et al., 2021 [299]** | Cohort study with retrospective control | lumbar degenerative disease | MIS-TLIF | 3.13d ±1.53 | Yes | Not reported | Fair |
| | | | | 3.71d ± 2.07 | | | |
| **Kilic et al., 2020 [174]** | Cohort study with retrospective control | lumbar scoliosis, degenerative spondylolisthesis, spinal canal stenosis | (hemi-)laminectomy, discectomy, fusion | 31.24 ± 4.87hours | Yes | 10 (11.6%)** | Good |
| | | | | 49.52 ± 5.96 hours | | 13 (14.8%)** | |
| **Lampilas et al., 2021 [88]** | Cohort study with retrospective control | NS | fusion surgery, cervical laminectomy, lumbar discectomy («lumbar discal hernia”) | 3.3d ± 0.9 | No | Not reported | Good |
| | | | | 6d ± 2.9 | | | |
| **Lei et al., 2023 [184]** | Cohort study with retrospective control | metastatic epidural spinal cord compression | decompression surgery (with transpedicular screw implantation & internal fixation) | 5.57d ± 2.52 | No | 8 (9.3%) | Good |
| | | | | 8.27d ± 3.98 | | 21 (21.43%) | |
| **Leng et al., 2022 [143]** | Cohort study with retrospective control | cervical spondylosis, cervical spondylotic myelopathy and radiculopathy | ACDF | median4d (3-11) | No | 10 (14.3%) | Fair |
| | | | | Median 5d (3-8) | | 32 (43.8%) | |

| | | | | | | | |
|---|---|---|---|---|---|---|---|
| **Li et al., 2018 [224]** | Cohort study with retrospective control | degenerative multilevel spine compression, cervical stenosis | laminoplasty | 5.75±2.46d | Yes | 24 (21.05%) | Good |
| | | | | 7.67±3.45 | | 23 (20.91%) | |
| **Liang et al., 2024** | RCT | lumbar spinal stenosis | NS | Not reported | Yes | *0 (0%)* | Fair |
| | | | | | | 5 (12.5%) "adverse reactions*"* | |
| **Liu et al., 2020 [94]** | RCT | intraspinal tumour | decompression &/ resection &/ stabilisation | Median 5 (3-16) | Yes | 4.4% | Good |
| | | | | Median 8 (3-22) | | 5.5% | |
| **Lovecchio et al., 2021 [82]** | Cohort study with retrospective control | adult spinal deformity | long-level (3-5) lumbar fusion to pelvis | 6.5 + 3.2 d | No | LOS<6d: 7 (20.1%) | Good |
| | | | | | | LOS>6d: 20 (41.5%) | |
| **Lu et al., 2023 [426]** | Cohort study with retrospective control | lumbar disc herniation, spondylolisthesis, Lig. flavum hypertrophy, facet arthritis (all diagnosed upon radiological manifestation) | microdiscectomy | 3d (2-4) | Yes | 8 (2.8%) | Good |
| | | | | 4d (3-4) | | 4 (2.9%) | |
| **Ma et al., 2024 [304]** | Cohort study with retrospective control | metastatic epidural spinal cord compression | decompression & stabilisation (posterolateral approach to circumferential decompression of vertebral canal, partial intralesional excision of metastatic spine tumour, spine stabilisation) | 6d (4-8) | Yes | 18 (13.04%) | Fair |
| | | | | 9d (6-11) | | 39 (23.49%) | |
| **Monk et al., 2023 [215]** | Case series | NS | MIS TLIF | 13.5 ± 6.3 hours | No | 0 (0%) | Fair |
| **Naik et al., 2023 [409]** | Cohort study with retrospective control | NS | fusion surgery | median 4.0d (3.0, 5.0) | No | 3 (5.6%)* | Good |
| | | | | median 2.0d (1.0 -3.75) | | 6 (11.1%)* | |
| **Nakamura et al., 2024 [54]** | Cohort study with retrospective control | Neuromuscular scoliosis | Posterior fusion | 20d (7 – 38) | No | 9 (33.33%) | Poor |
| | | | | 27d (10-113) | | 19 (70.37%) | |
| **Porche et al., 2022 [114]** | Cohort study with retrospective control | spondylolisthesis, spinal stenosis, nerve root compression, recurrent disc herniation, pseudoarthrosis, | TLIF | 3.6d ± 1.6 | Yes | 1.1 ± 1.1 | Good |

| | | | | | | | |
|---|---|---|---|---|---|---|---|
| | | adjacent segment disease | | | | | |
| | | | | 4.6d ± 1.7 | | 1.4 ± 1.1 | |
| **Porche et al., 2023 [366]** | Cohort study with retrospective control | cervical stenosis, OPLL, instability, nerve root compression, disc herniation, pseudoarthrosis, adjacent-segment disease | cervical laminoplasty & PCDF | 3.2d ± 1.4 | Yes | 1.1 ± 1.5* | Good |
| | | | | 4.7d ± 2.8 | | 1.8 ± 2.0* | |
| **Smith et al., 2019, [230]** | Cohort study with retrospective control | NS | Lumbar spine fusion | 92.3 ± 36.9hours | Yes | 7 (7.29%) | Good |
| | | | | 96.2 ± 32hours | | 2 (1.6%) | |
| **Soffin et al., 2019 [33]** | Consecutive case series | NS | ACDF, CDA | Median 416 min (210–1643) at PACU | No | 3 (9.1%) | Good |
| **Soffin et al., 2019 [36]** | Cohort study with retrospectively matched-controls (different anaesthetic protocols) | NS | Lumbar laminectomy / laminotomy &/ microdiscectomy | Median 237min (174-312min) for OFA vs. 274min (204-403min) for OCA | Yes | Not reported | Poor |
| **Soffin et al., 2019 [61]** | Retrospective cohort study, prospectively collected data | NS | lumbar laminotomy / laminectomy, lumbar microdiscectomy | Median 279 min (195 - 398) at PACU | No | 4 (6.5%) | Fair |
| **Soffin et al., 2020 [51]** | RCT | NS | lumbar fusion | 2.8 d (IQR 2.1-3.7) | Yes | 8 (36%) Cumulative risk 11,43% | Good |
| | | | | 3.1d(IQR2.8-4.8) | | 7 (35%) Cumulative risk 13,29% | |
| **Staartjes et al., 2019 [2579]** | Consecutive case series, no control | lumbar disc herniation, spondylolisthesis, stenosis, facet cysts, degenerative disc disease | Minimally invasive surgery | 1.1 ± 1.2 d | Yes | adverse events 95 (4%) (not further specified) | Good |
| **Stanton et al., 2024 [42]** | Cohort study retrospective controls, prospectively collected data | stenosis, spondylolisthesis, spondylosis, disc herniation, myelopathy, radiculopathy | decompression & revision surgery, fusion surgery | Median 2d (1-4.2) | Yes | Not reported | Poor |
| | | | | Median 3d (2-7) | | | |

| | | | | | | | |
|---|---|---|---|---|---|---|---|
| **Temby et al., 2021 [93]** | Cohort study with retrospective control | adolescent idiopathic scoliosis | posterior fusion surgery | 3.5d ± 0.9 | No | 3 (4.9%) | Good |
| | | | | 6.3d ± 0.9 | | 2 (6.3%) | |
| **Tondevold et al., 2021 [108]** | Cohort study with retrospective control | adolescent idiopathic scoliosis | Posterior spinal fusion | 3.6d | Yes | Not reported | Good |
| | | | | 6.3d | | | |
| **Tondevold et al., 2023 [46]** | Cohort study with retrospective control | neuromuscular scoliosis | posterior fusion | 6.5d | No | Not reported | Fair |
| | | | | 11d | | | |
| **Turcotte et al., 2021 [177]** | Retrospective observational cohort study | NS | posterolateral lumbar fusion | 2.12d ± 1.27 | No | Not reported | Fair |
| | | | | 2.60d ± 1.72 | | | |
| **van Hoorick et al., 2022 [25]** | Cohort study with retrospective controls, prospectively collected data | adolescent idiopathic scoliosis | posterior fusion surgery | 7.4d | Yes | Not reported | Fair |
| | | | | 7.5d | | | |
| **Venkata et al., 2018 [246]** | Consecutive case series | degenerative cervical / lumbar conditions causing neural compression | laminectomy, discectomy, cystectomy, nerve decompression | 12 patients (5%) staying longer than 24hours, 225 (95%) around 24h / ambulatory | No | Not reported | Fair |
| **Wang et al., 2017 [42]** | Consecutive case series | spondylolisthesis, severely degenerated disc with nerve root impairment | lumbar fusion | 1.29 ± 0.9 nights | No | 7 (16.67%) | Good |
| **Wang et al., 2020 [192]** | Cohort study with retrospective control | lumbar disc herniation, lumbar spinal stenosis | lumbar fusion | 12.30d ± 3.03 | Yes | 3 (3.125%) | Good |
| | | | | 15.50d ± 1.88 | | 9 (9.375%) | |
| **Wang et al., 2022 [54]** | Cohort study with retrospective control | lumbar disc herniation, lumbar spinal stenosis | long-level (≥3) lumbar fusion | 17.74d± 5.56 | No | 6 *(8.3%)* | Good |
| | | | | 22.13d ±12.21 | | 19 *(23.17%)* | |
| **Wang et al., 2024 [164]** | Study protocol | Degenerative spinal disorder | Spinal fusion in elderly patients | intended | None | intended | |

| | | | | | | | |
|---|---|---|---|---|---|---|---|
| **Wang et al., 2024 [245]** | Prospective cohort study (frail vs. non-frail) | Lumbar spinal stenosis, degenerative disc disease, lumbar spondylolisthesis | TLIF | Median 14d (12 - 18) | No | 30 (24.8%) | Good |
| | | | | Median 16d (13 – 21) | | 45 (36.3%) | |
| **Yao et al ., 2025 [130]** | Prospective cohort study (non-randomised; ERAS vs. ERAS with pre-rehab) | Degenerative spinal disease | Posterior lumbar spinal fusion | available | Yes | No | Poor |
| | | | | | | | |
| **Yokoi et al., 2024 [437]** | Cohort study with retrospective control | degenerative disease, tumour, revision fusion, infection | Pedicle subtraction osteotomy, corpectomy for neoplastic disease, instrumented fusion | 6d | No | Not reported | Fair |
| | | | | 7d | | | |
| **Young et al., 2021 [243]** | Cohort study with retrospective control | NS | cervical & lumbar fusion or decompression surgery | 51 ± 30 hours | No | 7 (7%) | Good |
| | | | | 62 ± 49 hours | | 9 (6%) | |
| **Yuan et al., 2023 [108]** | Cohort study with retrospective control | degenerative spinal deformity | posterior thoracolumbar fusion | 12d ± 4.5 | No | 12 (22.2%) | Good |
| | | | | 15.1d ± 7.7 | | 18 (33.3%) | |
| **Yuh et al., 2024 [7143 surgeries of 6350 patients]** | Cohort study with retrospective controls (ERAS 2021) | Primary spine tumours, degenerative spinal disease, "other" | MIS if possible; fusion surgery, discectomy, decompressive surgery | 3.37d | No | 41 (0.8% | Fair |
| | (ERAS 2003) | | | 7.38d | | 40 (2.4%) | |
| **Zhang et al., 2023 [70]** | RCT | Congenital scoliosis | posterior hemivertebra resection & spinal fusion | 6.4d ± 1.0 | Yes | 8 (22.9%) | Good |
| | | | | 7.2d ± 1.5 | | 14 (40%) | |
| **Zhong et al., 2021** | Study protocol | Cervical disc herniation with spinal cord compression (DCM-like) | ACDF | - | intended | intended | |

*Please note: the upper values are always for the ERAS cohort, the lower values for TDC cohorts; for studies comparing two ERAS cohorts at different points in time, the more recent ERAS cohort is represented by the top line (e.g. ERAS 2021 in upper row, ERAS 2003 in lower row)*

NA = not applicable
NS = not specified
NPC = «no pathway care»; patient undergoing surgery concurrent with ERAS cohort, but usually due to administrative reasons not undergoing a ERAS care
TLIF = transforaminal lumbar interbody fusion
PLF = posterolateral fusion
OLIF = oblique lumbar interbody fusion
* LOS on intensive care unit (ICU)
** 30d complications

§ costs as reported in study; for further evaluation they were later transformed into USD with regards to publication date & inflation
# study quality was assessed using the NHLBI study quality assessment tool
